# Analysis of Metabolic Profiles and Antioxidant Activity of Chinese Cordyceps, *Ophiocordyceps sinensis,* and *Paecilomyces hepiali* Based on Untargeted Metabolomics

**DOI:** 10.3390/biology13090683

**Published:** 2024-08-31

**Authors:** Min He, Chu-Yu Tang, Tao Wang, Meng-Jun Xiao, Yu-Ling Li, Xiu-Zhang Li

**Affiliations:** State Key Laboratory of Plateau Ecology and Agriculture, Qinghai Academy of Animal and Veterinary Science, Qinghai University, Xining 810016, China; himi1228@163.com (M.H.); chuyutang0410@163.com (C.-Y.T.); 13085500761@163.com (T.W.); 15574237597@163.com (M.-J.X.); yulingli2000@163.com (Y.-L.L.)

**Keywords:** untargeted metabolomics, chinese cordyceps, *ophiocordyceps sinensis*, *paecilomyces hepiali*, LC-MS/MS, antioxidant activity

## Abstract

**Simple Summary:**

Chinese cordyceps is a traditional medicinal fungus, *Ophiocordyceps sinensis*, and *Paecilomyces hepiali* are fungi isolated from wild Chinese cordyceps. These three species share similar chemical composition and pharmacological effects. This study utilized untargeted metabolomic technology (LC-MS/MS) to analyze metabolites in three samples. The antioxidant activity of the three species was evaluated using 2,2-diphenyl-1-picrylhydrazyl radical scavenging ability (DPPH•), ferric ion-reducing antioxidant power (FRAP), hydroxyl free radical scavenging capacity (•OH), and superoxide anion radical scavenging capacity (O_2_^•−^) to understand the differences in antioxidant activities among them. This study aimed to understand the metabolite differences and antioxidant differences of Chinese cordyceps, *P. hepiali*, and *O. sinensis*, and to reveal the roles of the differential metabolites in the antioxidant protection mechanism.

**Abstract:**

Chinese cordyceps (GL) is a traditional medicinal fungus, with *Ophiocordyceps sinensis* (*O. sinensis*, BL) and *Paecilomyces hepiali* (*P. hepiali*, JSB) being fungi isolated from wild Chinese cordyceps. These three species share similar chemical composition and pharmacological effects. Existing studies have primarily compared the metabolites of Chinese cordyceps and *O. sinensis*, overlooking the assessment of antioxidant capacity in Chinese cordyceps, *P. hepiali*, and *O. sinensis*. In this study, LC-MS/MS was employed to analyze metabolites in GL, JSB, and BL. Utilizing principal component analysis (PCA), supervised orthogonal partial least squares discriminant analysis (OPLS-DA), and hierarchical cluster analysis (HCA), it was observed that the majority of differential metabolites (DMs) primarily accumulated in organic acids and derivatives, lipids and lipid-like molecules, and organoheterocyclic compounds. Antioxidant activity analysis indicated that GL exhibited the higher 2,2-diphenyl-1-picrylhydrazyl radical scavenging ability (DPPH•, scavenging rate is 81.87 ± 0.97%), hydroxyl free radical scavenging capacity (•OH, scavenging rate is 98.10 ± 0.60%), and superoxide anion radical scavenging capacity (O_2_^•−^, scavenging rate is 69.74 ± 4.36%), while JSB demonstrated the higher FRAP total antioxidant capacity of 8.26 μmol Trolox/g (*p* < 0.05). Correlation analysis revealed a positive correlation between DMs (fatty acyls and amino acids) and DPPH•, FRAP, •OH, and O_2_^•−^ (*p* < 0.05). Additionally, glycerophospholipid DMs were found to be positively correlated with FRAP (*p* < 0.05). Through KEGG pathway analysis, it was determined that the accumulation of DMs in pathways such as cutin, suberine and wax biosynthesis has a higher impact on influencing the antioxidant activity of the samples. These results shed light on the antioxidant capacity and metabolic characteristics of Chinese cordyceps and its substitutes and offer valuable insights into how different DMs impact the strength of antioxidant activity, aiding in the advancement and application of Chinese cordyceps and its substitutes.

## 1. Introduction

Chinese cordyceps is a unique complex of fungi and host larvae that are transformed by *Ophiocordyceps sinensis* after infecting the larvae of *Hepialidae* Lepidoptera, utilizing the host larvae as its primary source of nutrition [1]. Numerous studies have demonstrated that Chinese cordyceps contain a variety of active ingredients, including protein, nucleotides, cordycepic acid, ergosterol, and polysaccharides [2]. It plays a crucial role in nourishing the kidneys and lungs, immune regulation, anti-aging, and antioxidant properties [3,4,5,6]. The nutritional and medicinal value of Chinese cordyceps is widely acknowledged by consumers, leading to a growing demand. However, Chinese cordyceps are primarily found in the Qinghai-Tibet Plateau and its surrounding regions, making artificial cultivation extremely challenging [7,8,9]. Uncontrolled harvesting has resulted in significant damage to the local ecosystem, leading to a depletion of wild Chinese cordyceps resources [10]. The limited availability of Chinese cordyceps has prompted the use of modern biological fermentation technology to cultivate Cordyceps mycelium as a substitute for natural Chinese cordyceps [11].

Two fungi, *Ophiocordyceps sinensis* [12,13] and *Paecilomyces hepiali* [14], have been identified in Chinese cordyceps. Research has indicated that these fungi share similar active compounds with Chinese cordyceps, including adenosine, polysaccharides, and sterols [15,16]. The closest genetic relationship between *O. sinensis* and Chinese cordyceps was revealed by analysis of fungal Internal Transcribed Spacer (ITS) fragment deoxyribonucleic acid (DNA) sequence and Random Amplified Polymorphic DNA (RAPD) [17,18,19]. This study provided evidence that *O. sinensis* is an asexual fungus of Chinese cordyceps [20,21]. *O. sinensis* is capable of counteracting the immunosuppressive effects of cyclophosphamide and enhancing the antioxidant capacity in immunosuppressed mice [22], and *O. sinensis* polysaccharide exhibits specific immunological activity [23]. The antihypertensive properties of the hot water extract of *P. hepiali* through experiments conducted on spontaneously hypertensive rats [24], and exhibited notable inhibitory effects on various cancer cell lines such as MCF-7, Hela, A549, and HepG2 [25,26,27,28]. Additionally, the health products “Bailing” capsules, made from *O. sinensis* powder obtained through liquid-submerged fermentation, and “Jin shui bao” capsules, made from *P. hepiali* mycelium powder, are popular in the market [29]. These studies reveal potential connections and similarities between these fungi and demonstrate that *O. sinensis* and *P. hepiali* are effective alternatives to Chinese cordyceps.

Antioxidants, short for antioxidant free radicals, are substances that effectively inhibit the oxidation reaction of free radicals. They work by directly removing or inhibiting oxidative free radicals and indirectly consuming substances that easily generate them, thus preventing further reactions [30]. The most prevalent free radicals in biological systems are oxygen-derived free radicals, also known as reactive oxygen species (ROS) [31]. When ROS is synthesized in the human body at a moderate level, it plays a beneficial physiological role [32,33,34]. However, an excessive amount of ROS in the body, leading to loss of control, disrupts the balance between antioxidant free radicals’ production and removal [35]. This imbalance can result in damage to various biological macromolecules in the body, such as lipid peroxidation, cross-linking or breakage of membrane proteins and DNA [36,37,38], accelerating the aging process and potentially contributing to diseases like cancer, diabetes, rheumatoid arthritis, and Alzheimer’s disease, among others [39]. Consequently, enhancing the body’s antioxidant capacity through the consumption of exogenous antioxidants has emerged as an effective strategy to alleviate oxidative stress and prevent a range of diseases [40]. Chinese cordyceps, as a special “medicine and food homology” fungi [41], has a variety of pharmacological effects such as antioxidant, anti-inflammatory and immunomodulatory effects [3,4,5,6]. Therefore, the consumption of Chinese cordyceps helps humans to reduce problems such as oxidative stress.

Chinese cordyceps are rich in active components such as phenols, lipids, and nucleosides, which play an important role in scavenging ROS [2]. Research has indicated that Chinese cordyceps polysaccharides extend the lifespan of Drosophila by upregulating the expression levels of antioxidant-related genes such as CAT, SOD, and MTH [42]. The D-mannitol of Chinese cordyceps possesses the ability to eliminate hydroxyl free radicals and superoxide anion free radicals [43,44,45]. Chinese cordyceps alleviates oxidative stress in mice with pulmonary fibrosis by increasing the activity of SOD and GSH-Px [46]. The adenosine of Chinese cordyceps through mice experimentally demonstrated largely abrogates oxidative stress and damage in glucose or pyruvate-perfused hearts [47]. The polyphenolics of Chinese cordyceps have an effect on alleviating protein oxidation and low-density lipoprotein oxidation [48]. Therefore, in addition to investigating the antioxidant properties of wild Chinese cordyceps and artificial Chinese cordyceps, it is imperative to delve into the study of their fermentation mycelium. However, existing studies have mainly focused on the pharmacological effects of the active ingredients, and the potential differences between these strains need to be further investigated.

Previous studies have highlighted variations in metabolites between Chinese cordyceps and *O. sinensis* [49]. However, there is a limited amount of research comparing the metabolomics and antioxidant capacity of Chinese cordyceps, *P. hepiali*, and *O. sinensis*. This study utilized high-performance liquid chromatography-tandem mass spectrometry (LC-MS/MS) metabolomics technology to perform untargeted metabolomic analysis of metabolites in Chinese cordyceps, *P. hepiali*, and *O. sinensis*. Differential metabolites of 3 samples were assessed through principal component analysis (PCA), supervised orthogonal partial least squares discriminant analysis (OPLSDA), hierarchical cluster analysis (HCA), and Pearson correlation coefficient. The antioxidant activity of Chinese cordyceps, *P. hepiali*, and *O. sinensis* was evaluated using 2,2-diphenyl-1-picrylhydrazyl radical scavenging ability (DPPH•), ferric ion-reducing antioxidant power (FRAP), hydroxyl free radical scavenging capacity (•OH), and superoxide anion radical scavenging capacity (O_2_^•−^) to understand the differences in antioxidant activities among them. Furthermore, a correlation analysis between the identified differential metabolites and the four antioxidant indicators was conducted. The goal is to determine if there is a relationship between these differential metabolites and antioxidant properties and to understand their possible role in increasing or decreasing antioxidant activity. In summary, this study aimed to understand the metabolite differences and antioxidant differences of Chinese cordyceps, *P. hepiali*, and *O. sinensis*, and to reveal the roles of the differential metabolites in the antioxidant protection mechanism, to provide the theoretical basis for the development of novel and efficient antioxidants.

## 2. Materials and Methods

### 2.1. Materials

**Figure 1:**
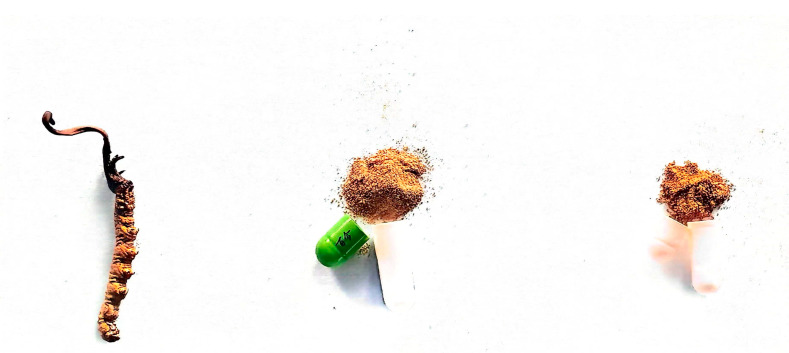
The specimens of Chinese cordyceps and its substitutes. On the left is Chinese cordyceps (GL), in the middle is *Ophiocordyceps sinensis* (BL), and on the right is *Paecilomyces hepiali* (JSB).

The Chinese cordyceps (33°40′34″ N, 98°57′07″ E, altitude: 4238 m) with a small amount of soil were hand-dug, stored in dry ice and transported. The soil was cleaned at room temperature, and Chinese cordyceps were washed with deionized water three times and dried naturally in the shade. Chinese cordyceps, *O. sinensis*, and *P. hepiali* with the weight of 2 g were loaded into EP tubes, respectively. They were stored at room temperature for use. Chinese cordyceps were purchased from Qinghai Baohuitang Biotechnology Co., Ltd. (Xining, China). *O. sinensis* and *P. hepiali* were purchased from pharmacies. All samples are dry; see Figure 1 and Table 1 for specific information.

### 2.2. Untargeted Metabolomics Profiling

#### 2.2.1. QC Sample Preparation and Metabolites Extraction

The low-temperature high-speed centrifuge with 5430R (Eppendorf, Hamburg, Germany) and Ultrasonic Instrument with KQ-250DE (Kunshan Ultrasonic Instrument Co., Ltd., Kunshan, China) were used in the study. Ammonium acetate (NH_4_AC, purity ≥ 99%) from Sigma Aldrich (Saint Louis, MO, USA), acetonitrile (chromatographically pure, LC-MS grade) from Merck (Darmstadt, Germany), and ammonium hydroxide (NH_4_OH, purity ≥ 99%) and methanol (chromatographically pure, LC-MS grade) from Fisher (Waltham, MA, USA).

Grind all Chinese cordyceps, *O. sinensis*, and *P. hepiali* into a fine powder using a mortar and pestle, respectively. Add 0.1 g of powder from each of the 3 samples after passing through a 40-mesh sieve to a 1000 μL mixed solution (methanol:acetonitrile:aqueous = 2:2:1 (*v*/*v*)). Vortex the solution for 30 s to ensure homogeneity, ultrasonic at ice water for 30 min, standing at −20 °C for 10 min, centrifuge at 14,000× *g* and 4 °C for 20 min, take 500 μL of the supernatant and dry it under vacuum. Six replicates were performed for each sample. In mass spectrometry analysis, the 18 dried samples were redissolved by adding them to 100 μL of acetonitrile solution (acetonitrile:water = 1:1, *v*/*v*), respectively. Then, swirl for 30 s, sonicate in ice water for 30 min, and centrifuge at 14,000× *g* and 4 °C for 15 min. Finally, 120 μL of the supernatant was used for LC-MS/MS analysis. Take 10 μL from each of the three samples and combine into a quality control sample (QC). Before the formal testing, the QC sample should be inserted to assess the instrument status and calibrate the chromatography–mass spectrometry system. This step is crucial for evaluating system stability and ensuring data reliability throughout the experiment. 

#### 2.2.2. LC-MS/MS Condition

The samples were separated by Agilent 1290 Infinity LC ultra-high performance liquid chromatography (Agilent, Santa Clara, CA, USA) HILIC column. Column temperature 25 °C; flow rate 0.5 mL/min; sample size 2 μL; mobile phase composition A: water + 25 mM ammonium acetate + 25 mM ammonia water, B: acetonitrile. The samples were placed in a 4 °C automatic injector during the entire analysis process. To avoid the influence caused by the fluctuation of the instrument detection signal, the samples are analyzed continuously in random order. The gradient separation procedure is shown in Table 2.

#### 2.2.3. Mass Spectrum Condition

The AB Triple TOF 6600 mass spectrometer (Shanghai Applied Protein Technology Co., Ltd., Shanghai, China) was used to collect the primary and secondary spectra of the samples. The positive and negative ESI Source conditions after HILIC chromatographic separation are as follows: Ion Source Gas1 (Gas1): 60, Ion Source Gas2 (Gas2): 60, Curtain gas (CUR): 30, source temperature: 600 °C, IonSapary Voltage Floating (ISVF) ±5500 V; TOF MS scan *m*/*z* range: 60–1000 Da, product ion scan *m*/*z* range: 25–1000 Da, TOF MS scan accumulation time 0.20 s/spectra, product ion scan accumulation time 0.05 s/spectra; sheath gas flow rate, 35 arbitrary units; auxiliary gas flow rate, 8 arbitrary units. Secondary mass spectrometry was obtained using information-dependent acquisition (IDA) and high sensitivity mode, Declustering potential (DP): ±60 V (positive and negative modes), Collision Energy: 35 ± 15 eV, IDA Settings are as follows: Exclude isotopes within 4 Da, Candidate ions to monitor per cycle: 10. 

#### 2.2.4. Data Processing

The raw MS data were converted to MzXML files using ProteoWizard MSConvert before being imported into freely available XCMS software (XCMS online 3.7.1). The following parameters were used for peak picking: cent Wave *m*/*z* = 10 ppm, peakwidth = c (10, 60), prefilter = c (10, 100). For peak grouping, bw = 5, mzwid = 0.025, and minfrac = 0.5 were used. CAMERA (Collection of Algorithms of MEtabolite pRofile Annotation) was used for annotating isotopes and adducts. In the extracted ion features, only the variables having more than 50% of the nonzero measurement values in at least one group were kept. Compound identification of metabolites was performed by comparing the accuracy *m*/*z* value (<10 ppm) and MS/MS spectra with an in-house database established with available authentic standards. The missing data were filled in by the KNN (K-Nearest Neighbor) method, and the extreme values were deleted. Finally, the total peak area of the data was normalized to ensure the parallelism between samples and metabolites. 

### 2.3. Determination of Antioxidant Activity

The DPPH• kit, FRAP kit, and •OH kit were procured from Suzhou Keming Biotechnology Co., Ltd. (Suzhou, China), while the O_2_^•−^ kit was obtained from Beijing Solarbio Technology Co., Ltd. (Beijing, China). Ascorbic acid (VC) was used as positive control for the 4 antioxidant assay indexes, and the experimental method for each assay index was carried out in strict accordance with the instructions.

#### 2.3.1. Determination of DPPH Free Radical Scavenging Ability

Determination of 2,2-diphenyl-1-picrylhydrazyl radical scavenging ability (DPPH•) by antioxidant capacity kit and some modifications were made based on previous work [50]. Weigh approximately 0.1 g samples from GL, BL, and JSB, and add 1 mL of anhydrous sodium acetate–glacial acetic acid solution (extraction solution) to them, respectively. Homogenize in an ice bath, and prepare 3 separate solutions with a concentration of 100 mg/mL each. Centrifuge at 10,000× *g* and 4 °C for 10 min and place on ice to be measured. Set a blank tube and add 50 μL extract and 950 μL reagent solution, each measuring tube was added with 50 μL sample solution and 950 μL reagent solution. Mix all the prepared solutions thoroughly, let them react in the dark for 20 min, and measure the absorbance value at 515 nm. The measuring tube of each sample is set up in 3 replicates, and the average value is obtained. The DPPH radical scavenging rate of the sample can be calculated using the formula:

DPPH free radical scavenging rate (%) = (A_0_ − A_1_) ÷ A_0_ × 100%

Among, A_0_ is the blank tube and A_1_ is the measurement tube.

#### 2.3.2. Determination of FRAP Total Antioxidant Capacity

Weigh approximately 0.1 g samples from GL, BL, and JSB, and add 1 mL of anhydrous sodium acetate–glacial acetic acid solution (extraction solution) to them, respectively. Homogenize in an ice bath, and prepare 3 separate solutions with a concentration of 100 mg/mL each. Centrifuge at 10,000× *g* and 4 °C for 10 min and place on ice to be measured. The mixed solution includes anhydrous sodium acetate–glacial acetic acid solution, TPTZ solution, and FeCl_3_·6H_2_O solution. Set a blank tube and add 50 μL anhydrous sodium acetate–glacial acetic acid and 950 μL mixed solution, each measuring tube was added with 50 μL sample solution and 950 μL mixed solution. Mix all the prepared solutions thoroughly, react at room temperature for 20 min, and measure the absorbance value at 593 nm. The measuring tube of each sample is set up in 3 replicates, and the average value is obtained. The total antioxidant capacity of FRAP can be calculated using the standard curve formula:

y = 2.4832x + 0.0134, R^2^ = 0.9996

Here, y is the absorbance value difference A (A = A_1_ − A_0_). A_0_ is the blank tube and A_1_ is the measurement tube. The variable x is the Trolox concentration (μmol/mL). The total antioxidant capacity of the sample is determined by the quantity of antioxidant Trolox measured from the standard curve.

#### 2.3.3. Determination of Hydroxyl Free Radical Scavenging Ability

Weigh approximately 0.1 g samples from GL, BL, and JSB, and add 1 mL of distilled water to them, respectively. Homogenize in an ice bath, and prepare 3 separate solutions with a concentration of 100 mg/mL each. Centrifuge at 10,000× *g* and 4 °C for 10 min and place on ice to be measured. Prepare a blank tube by adding 150 μL of salicylic acid–ethanol solution, 300 μL of H_2_O_2_ solution, and 900 μL of H_2_O. Add 150 μL of salicylic acid–ethanol solution and FeSO_4_·7H_2_O, 300 μL of H_2_O_2_ solution, and 750 μL of distilled water to a control tube. To each measuring tube, add 150 μL of salicylic acid–ethanol solution, 150 μL of FeSO_4_·7H_2_O, 450 μL of distilled water, and 300 μL of sample solution. Mix all prepared solutions thoroughly, incubate at 37 °C for 20 min, and measure the absorbance value at 510 nm. The measuring tube of each sample is set up in 3 replicates, and the average value is obtained. The hydroxyl radical scavenging rate of the sample can be calculated using the formula:

Hydroxyl free radical scavenging rate (%) = (A_1_ − A_2_) ÷ (A_1_ − A_0_) × 100%

Among, A_0_, A_1_, and A_2_ are the blank tube, control tube, and measurement tube, respectively.

#### 2.3.4. Determination of Superoxide Anion Radical Scavenging Capacity

Determination of superoxide anion radical scavenging capacity (O_2_^•−^) by antioxidant capacity kit and some modifications were made based on previous work [51]. Weigh approximately 0.1 g samples from GL, BL, and JSB, and add 1 mL of KH_2_PO_4_-K_2_HPO_4_·3H_2_O solution containing disodium EDTA (ethylenediaminetetraacetic acid) salt, TritonX-100, and polyvinyl pyrrolidone (PVP) to them, respectively. Homogenize in an ice bath, and prepare 3 separate solutions with a concentration of 100 mg/mL each. Centrifuge at 10,000× *g* and 4 °C for 10 min and place on ice to be measured. Prepare a blank tube by adding 40 µL of Tris-HCL solution, 100 µL of distilled water, 160 µL ammonium persulfate solution, 200 µL of hydroxylamine hydrochloride solution, 200 µL of P-amino benzene sulfonic acid-acetic acid solution, and 200 µL of alpha naphthylamine acetic acid solution. Each measuring tube was added with 40 µL of Tris-HCL solution, 100 µL of the sample solution, 160 µL ammonium persulfate solution, 200 µL of hydroxylamine hydrochloride solution, 200 µL of P-amino benzene sulfonic acid-acetic acid solution, and 200 µL of alpha naphthylamine acetic acid solution. Mix all prepared solutions thoroughly, develop color at 37 °C for 20 min, then transfer the solutions into a 1 mL glass cuvette and measure the absorbance value at 530 nm. The measuring tube of each sample is set up in 3 replicates, and the average value is obtained. The superoxide anion scavenging rate of the sample can be calculated using the formula:

Superoxide anion radical scavenging rate (%) = (A_0_ − A_1_) ÷ A_0_ × 100%

Among, A_0_ is the blank tube and A_1_ is the measurement tube.

### 2.4. Statistical Analyses

The raw data were processed based on information from the Kyoto Encyclopedia of Genes and Genomes database (KEGG, https://www.genome.jp/kegg/pathway.html, accessed on 15 March 2024) and the Human Metabolome Database (HMDB, http://www.hmdb/, accessed on 15 March 2024) for compound annotation. Unsupervised and supervised dimensionality reduction methods were utilized to conduct principal component analysis (PCA) and orthogonal partial least squares discriminant analysis (OPLS-DA) using the R package model (http://www.r-project.org/, accessed on 13 March 2024). Use the R pheatmap software (v1.0.12) [52] to create a hierarchical cluster (HCA) heatmap after the z-score normalization of metabolites in all samples. Differential metabolites were identified based on the variable weight value (VIP) and *p* value from the (O)PLS model, with metabolites having VIP > 1 and *p* < 0.05 considered as differential. Correlation and significance analysis of data on DMs and antioxidant activity were performed using SPSS 26.0 software. Data plots in Section 3.4 were generated using ChiPlot (https://www.chiplot.online/, accessed on 9 July 2024 [53]).

## 3. Results

### 3.1. Metabolite Profiles Analysis

**Figure 2:**
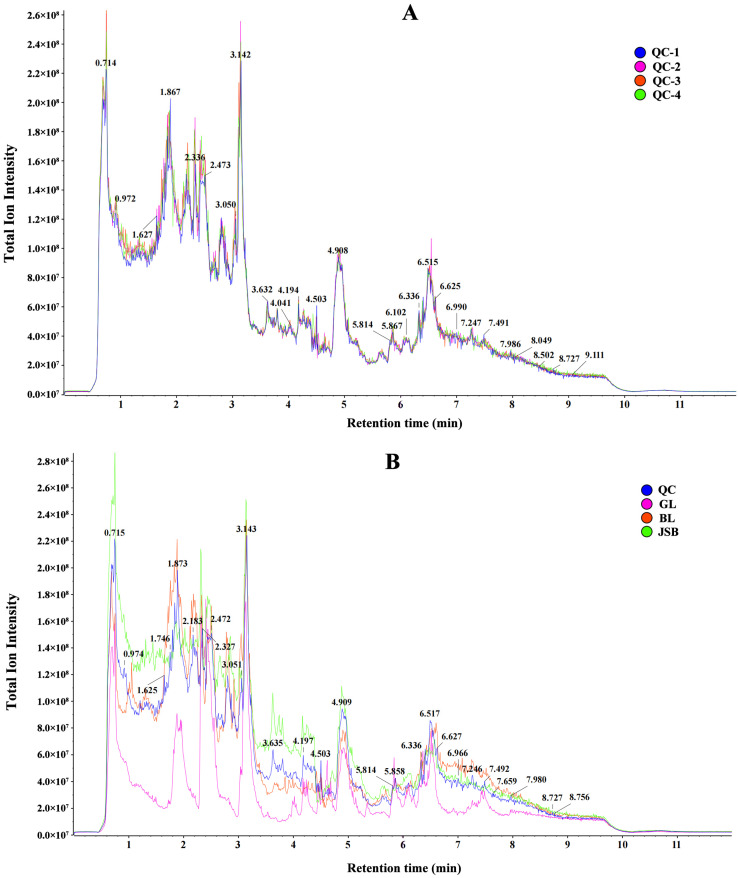
(**A**) Total ion chromatograms (TIC) of QC (quality control) with untargeted metabolomics LC-MS/MS technology. (**B**) TIC of QC, GL, BL, and JSB.

This study utilized LC-MS/MS technology for untargeted metabolomics to generate total ion chromatograms (TIC) of QC, GL, BL, and JSB. The results of QC retention time and peak intensity overlap showed that MS maintained a stable signal when probing the same sample at different times (Figure 2A). The peak heights and positions of GL, BL, and JSB showed significant variations within the 0.5–9 min time frame (Figure 2B).

**Figure 3:**
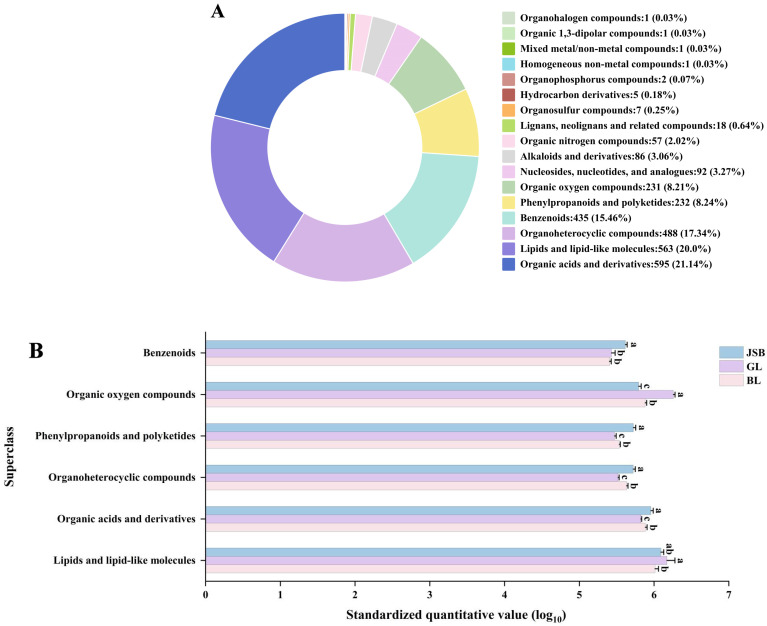
The metabolites were detected in GL, BL, and JSB. (**A**) Metabolic classification (superclass) of GL, BL, and JSB. (**B**) The quantitative value expression of the top 6 superclasses in the 3 samples. The ANOVA analysis shows that different lowercase letters indicate significant differences between samples at the 0.05 level.

To explore the metabolic profiles of Chinese cordyceps and its substitutes, LC-MS/MS technology was utilized in conjunction with public databases such as Mass Bank, Metlin, and MoNA, along with a proprietary secondary mass spectrometry database for the analysis of GL, BL, and JSB metabolisms. The 2814 known metabolites were identified from GL, BL, and JSB, comprising 1462 ESI+ metabolites and 1352 ESI− metabolites. These metabolites were categorized into 17 superclasses, which included organic acids and derivatives, lipids and lipid-like molecules, organoheterocyclic compounds, benzenoids, phenylpropanoids and polyketides, organic oxygen compounds, nucleosides, alkaloids and derivatives, organic nitrogen compounds, lignans and related compounds, and so on (Figure 3A). Among them, organic acids and derivatives have the highest number of metabolites at 20.14%, followed by lipids and lipid-like molecules, organoheterocyclic compounds, and benzenoids at 20.0%, 17.34%, and 15.46%, respectively.

The quantitative values of the top 6 superclasses in 3 samples were standardized for differential expression analysis (Figure 3B). The results showed that GL contained more organic oxygen compounds and lipids and lipid-like molecules (*p* < 0.05) than BL and JSB. The JSB contained more benzenoids, organoheterocyclic compounds, phenylpropanoids and polyketides, and organic acids and derivatives. It is worth noting that the expression of BL in these 6 superclasses is the second place.

**Figure 4:**
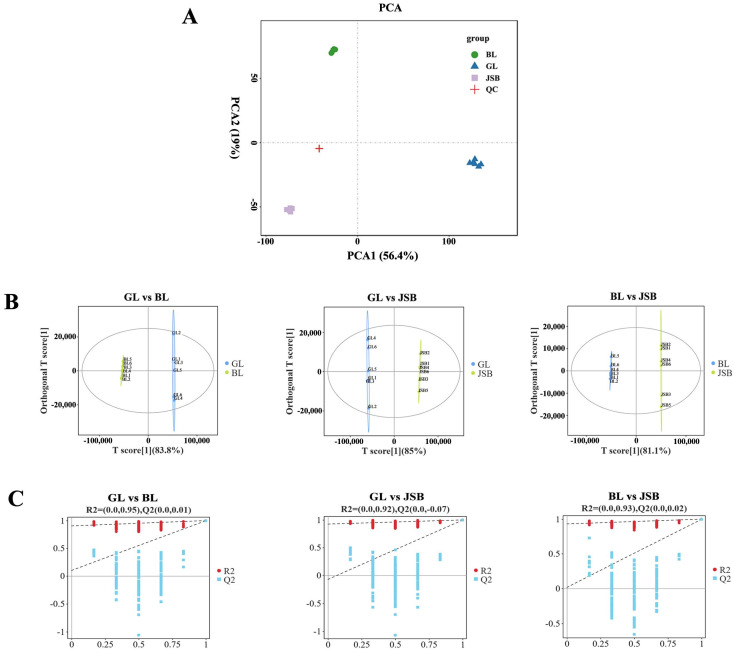
Multivariate statistical analyses of metabolites detected in GL, BL, and JSB. (**A**) PCA analyses of GL, BL, JSB, and QC. (**B**) OPLS-DA scores of GL, BL, and JSB. (**C**) OPLS-DA permutation test of GL, BL, and JSB.

Principal Component Analysis (PCA) was employed to analyze the metabolome of GL, BL, JSB, and QC. This analysis revealed distinct differences among the sample groups and the variability present within each group [54]. The PCA plot illustrates that PC1 accounts for 56.4% of the variance while PC2 accounts for 19%. There is strong aggregation and repeatability within each sample group, with clear separation trends observed among the samples in each group, suggesting significant differences in the compounds of GL, BL, and JSB. The QC samples clustered closely to the center of the PCA plot, indicating minimal deviation during the measurement process, thus confirming the stability of the method and the metabolome data for each sample (Figure 4A). The results of the OPLS-DA analysis demonstrate that both sample groups in each model fall within the confidence zone, with R^2^Y > 0.9 and Q^2^ > 0.9, indicating the reliability of the model (Figure 4B). The results of the permutation test reveal a gradual decrease in R^2^ and Q^2^ values as the permutation retention decreases. The Q^2^ intercepts from the permutation test for the three sample groups are 0.01, −0.07, and 0.02, all <0.5, suggesting the absence of overfitting in the original model and its good stability (Figure 4C).

### 3.2. Screening of Differential Metabolites (DMs)

**Figure 5:**
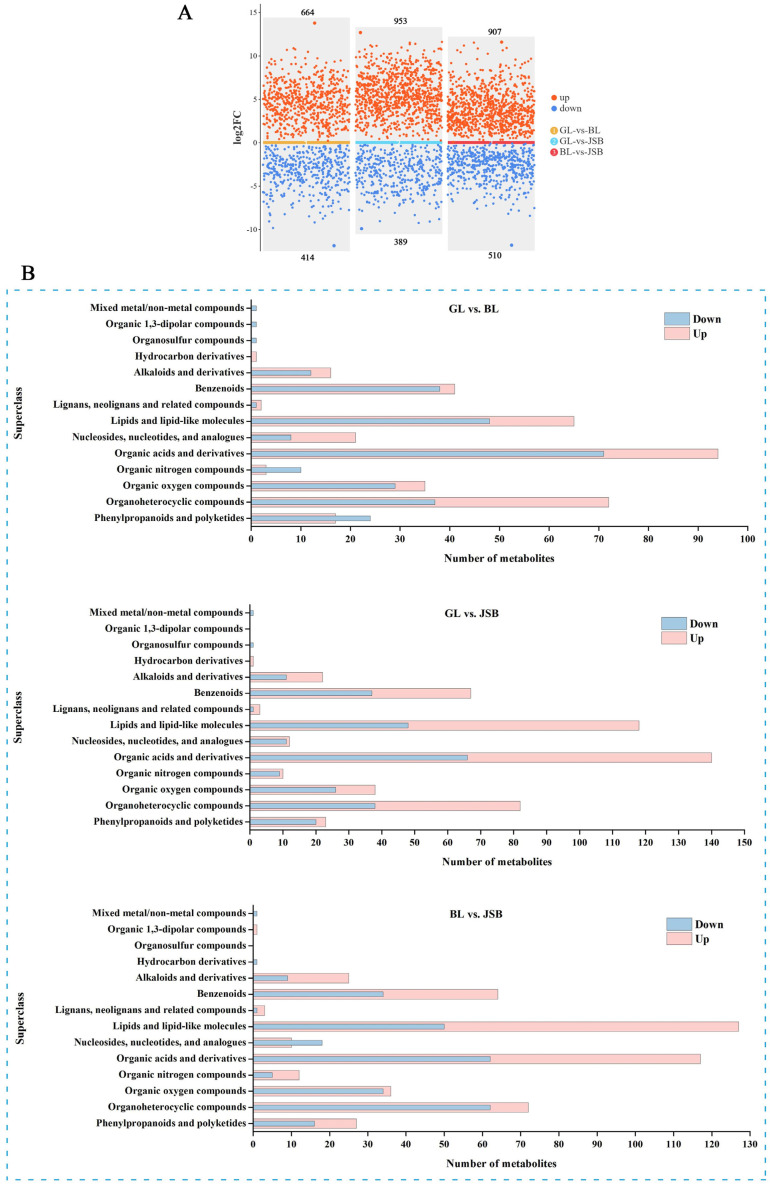
The DAMs analysis of the GL, BL, and JSB. (**A**) Difference scatter plots of GL, BL, and JSB. Red points indicate VIP ≥ 1 and *p* < 0.05, representing upregulated differential metabolites (log2_FC > 1); blue points indicate VIP ≥ 1 and *p* < 0.05, representing downregulated differential metabolites (log2_FC < −1). Larger points indicate higher VIP values of the metabolite, and the three colored bars in the middle represent the three comparison groups. (**B**) The superclass classification of DMs in the pairwise comparison between GL and BL, GL and JSB, and BL and JSB.

The VIP value of the first principal component in the OPLS-DA model serves as an indicator of the strength of influence of each metabolite content on the sample. The combination of t-test with a significance level of *p* < 0.05 and VIP > 1 is utilized to identify differential metabolites and auxiliary markers between different groups, aiding in the process of metabolite screening [55,56]. The scatter plot visualization results reveal that there are 1078 DMs (664 upregulated and 414 downregulated) between GL and BL, 1342 DMs (953 upregulated and 389 downregulated) between GL and JSB, and 1417 DMs (907 upregulated and 510 downregulated) between JSB and BL (Figure 5A). We found that BL exhibited significant upregulation of organoheterocyclic compounds, organic acids and derivatives, nucleosides and analogues, hydrocarbon derivatives, lipids compounds, and so on. GL contained a higher proportion of organic nitrogen compounds and phenylpropanoids and polyketides. JSB showed significant upregulation of organoheterocyclic compounds, organic oxygen compounds, organic acids and derivatives, lipids compounds, and so on. However, BL had higher quantities of hydrocarbon derivatives and nucleosides and analogues compared to JSB (Figure 5B).

**Figure 6:**
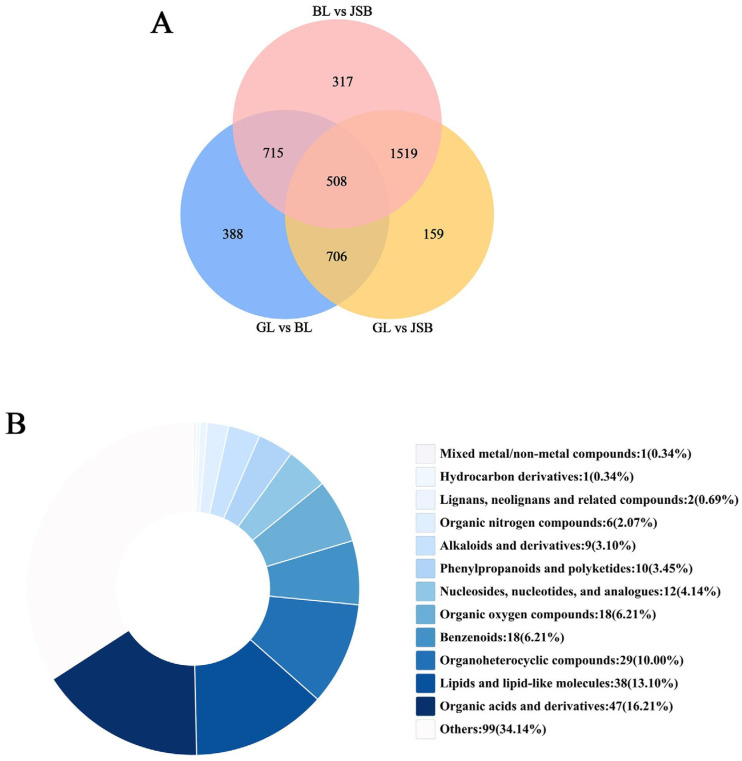
(**A**) Venn diagram of metabolites detected from GL and BL, GL and JSB, and BL and JSB. (**B**) Superclass classification of the 508 key metabolites.

The Venn diagram exhibits that 508 overlapping DMs among GL, BL, and JSB can be further categorized into 12 superclasses and 191 known DMs, including lipid compounds like oleic acid, azelaic acid, and acetylcarnitine, as well as amino acid compounds such as glutamic acid, L-arginine, and L-histidine (Figure 6A). More detailed information can be found in Table S1. The top three supercategories among these 12 superclasses are organic acids and derivatives, lipid compounds, and organoheterocyclic compounds (Figure 6B). Therefore, DMs belonging to these superclasses can serve as potential biomarkers for distinguishing GL, BL, and JSB.

### 3.3. DMs Cluster Analysis

**Figure 7:**
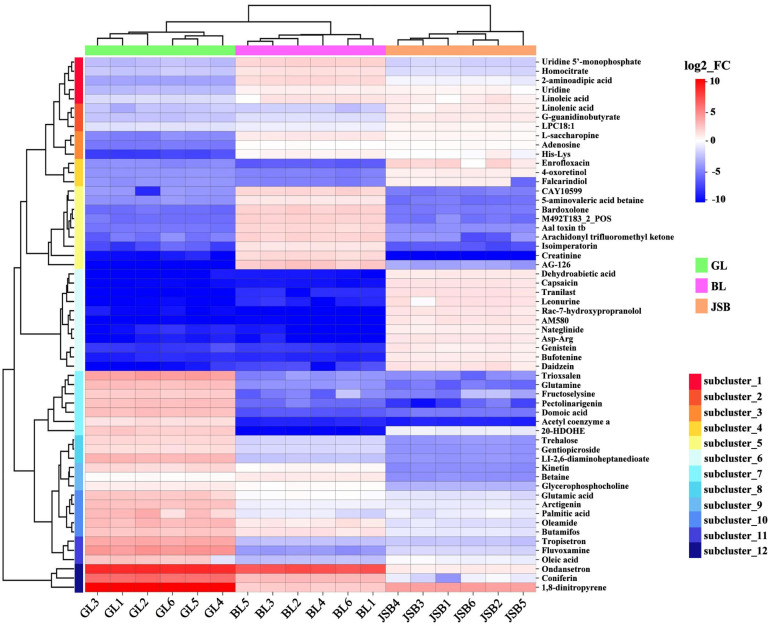
Top 58 hierarchical cluster analysis (HCA) between GL, BL, and JSB. Redder colors indicate higher metabolite abundance; bluer colors indicate lower metabolite abundance.

Among the numerous differential metabolites, we identified the top 58 metabolites with high VIP values for further visual analysis. Our results revealed that these metabolites can be categorized into 12 subclusters (Figure 7). The results showed that lipid metabolites (oleic acid, palmitic acid, oleamide, acetyl coenzyme a) and organic acid metabolites (domoic acid, fructoselysine, glutamic acid, glutamine, Ll-2,6-diaminoheptanedioate) were significantly expressed in the GL. JSB exhibited the highest expression abundance of lipid metabolites (Lpc 18:1, 4-oxoretinol, dehydroabietic acid, linolenic acid, falcarindiol) and organic acid metabolites (nateglinide, g-guanidinobutyrate, asp-arg). Additionally, creatinine and homocitrate showed higher expression abundance among the amino acids in BL. The DMs of GL, BL, and JSB predominantly reside within lipids and organic acids, offering valuable insights for identifying potential biomarkers.

### 3.4. Correlation Analysis

**Figure 8:**
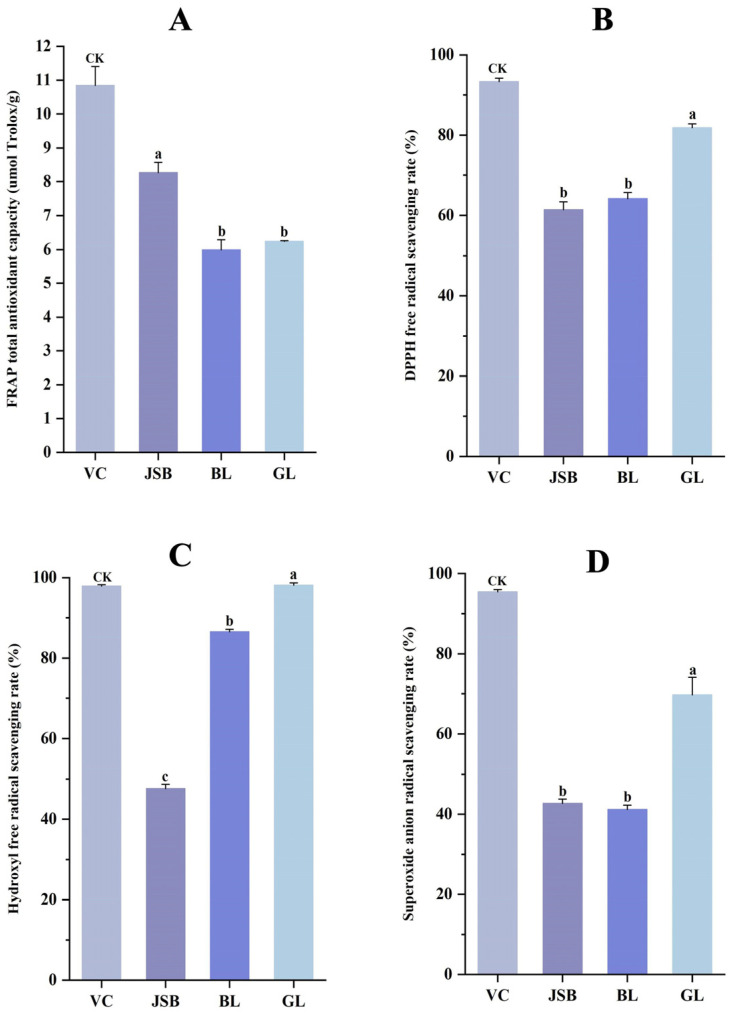
Comparison of antioxidant activity between GL, BL, and JSB. Ascorbic acid (VC) was a control sample and was abbreviated as CK. (**A**) Ferric ion-reducing antioxidant power (FRAP), (**B**) 2,2-diphenyl-1-picrylhydrazyl radical scavenging ability (DPPH•), (**C**) hydroxyl free radical scavenging capacity (•OH), (**D**) superoxide anion radical scavenging capacity (O_2_^•−^). The ANOVA analysis shows that different lowercase letters indicate significant differences between samples at the 0.05 level.

The antioxidant activities of GL, BL, and JSB were assessed using the DPPH•, FRAP, •OH, and O_2_^•−^ methods. Results indicate that GL exhibited the highest DPPH free radical scavenging rate (81.87 ± 0.97%), hydroxyl free radical scavenging rate (98.10 ± 0.60%), and superoxide anion radical scavenging rate (69.74 ± 4.36%), while JSB showed the highest FRAP total antioxidant capacity (8.26 μmol Trolox/g). No significant differences were observed in DPPH• and O_2_^•−^ between BL and JSB (*p* > 0.05). However, there were significant differences in •OH levels among the three samples (*p <* 0.05). The disparity in FRAP values between BL and GL was also not significant (*p* > 0.05) (Figure 8A–D).

**Figure 9:**
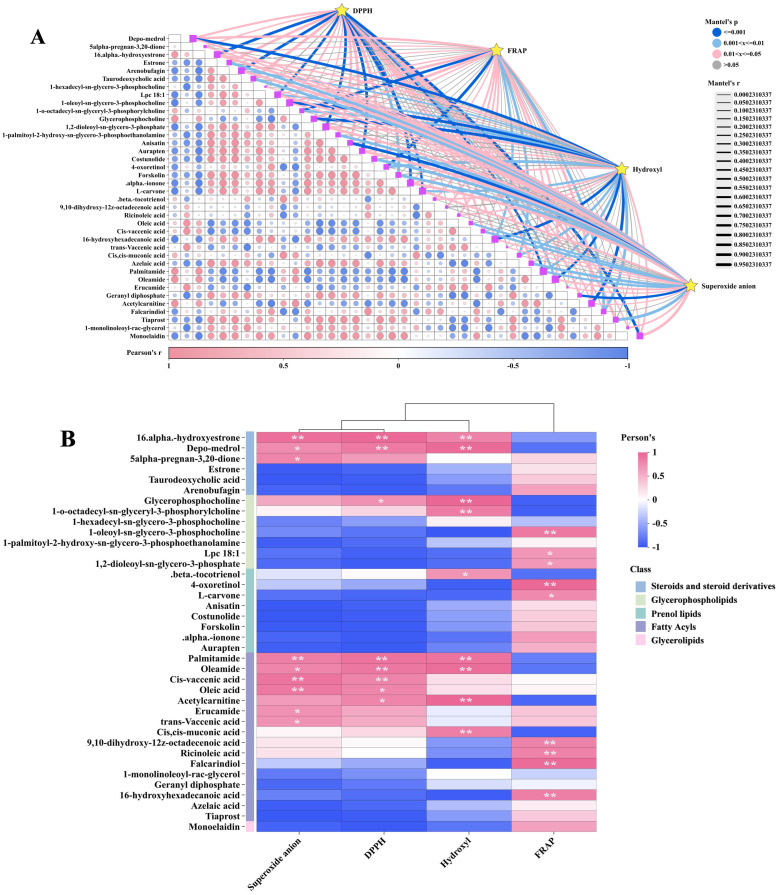
Comparison of antioxidant activity between GL, BL, and JSB. (**A**) Interactive Mantel test correlation heatmap analysis of DPPH•, FRAP, •OH, O_2_^•−^, and key lipid metabolites. (**B**) The inter-group correlation heatmap analysis of DPPH•, FRAP, •OH, O_2_^•−^, and key lipid metabolites. The vertical coordinate is DMs, and the horizontal coordinate is the 4 antioxidant indicators. **, *p* values < 0.01. *, *p* values < 0.05.

**Figure 10:**
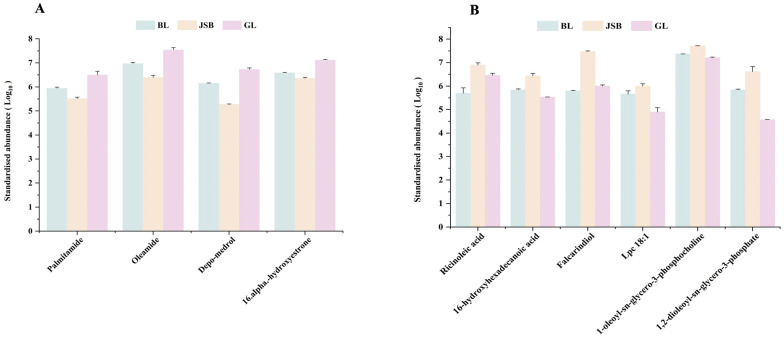
Comparison of key lipid metabolites content between GL, BL and JSB. (**A**) The DMs are significantly associated with the antioxidant activities of DPPH•, •OH, and O_2_^•−^. (**B**) The DMs are significantly associated with the antioxidant activities of FRAP.

Mantel test and Pearson correlation analysis were employed to investigate the relationship between DPPH•, FRAP, •OH, O_2_^•−^, and these key DMs. The Mantel test results indicated significant correlations between DPPH•, FRAP, •OH, and O_2_^•−^ with multiple lipid DMs (Figure 9A). The correlation heatmap illustrates that the DMs of most fatty acyls (e.g., palmitamide and oleamide) and steroids and steroid derivatives (e.g., depo-medrol and 16-α-hydroxyestrone) exhibit a positive correlation with DPPH•, •OH, and O_2_^•−^. Fatty acyls like ricinoleic acid, 16-hydroxyhexadecanoic acid, and falcarindiol, as well as glycerophospholipids including Lpc 18:1, 1-oleoyl-sn-glycero-3-phosphocholine, and 1,2-dioleoyl-sn-glycero-3-phosphate, are positively correlated with FRAP (Figure 9B). The abundance of these DMs is generally in line with the trends observed in DPPH•, FRAP, •OH, and O_2_^•−^ in various samples (Figure 10A,B).

**Figure 11:**
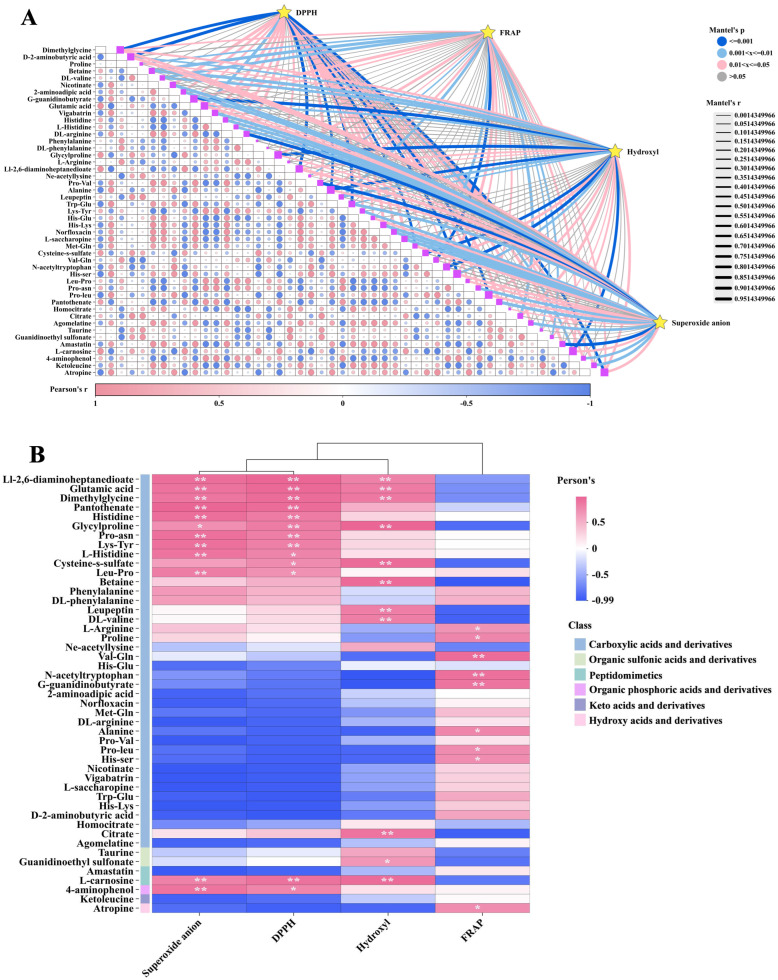
Comparison of antioxidant activity between GL, BL, and JSB. (**A**) Interactive Mantel test correlation heatmap analysis of DPPH•, FRAP, •OH, O_2_^•−^, and key organic acids and derivatives. (**B**) The inter-group correlation heatmap analysis of DPPH•, FRAP, •OH, O_2_^•−^, and key organic acids and derivatives. The vertical coordinate is DMs, and the horizontal coordinate is the 4 antioxidant indicators. **, *p* values < 0.01. *, *p* values < 0.05.

**Figure 12:**
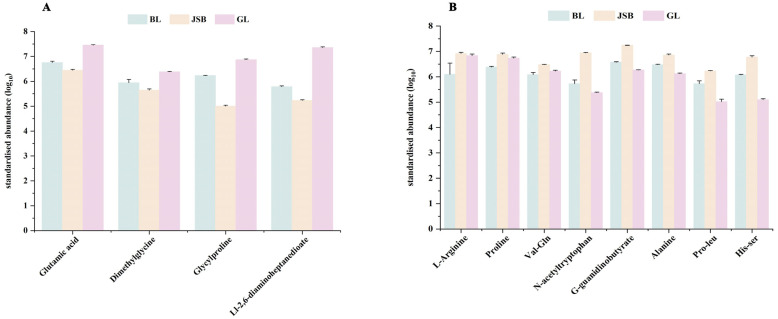
Comparison of key organic acids and derivatives content between GL, BL and JSB. (**A**) The DMs are significantly associated with the antioxidant activities of DPPH•, •OH, and O_2_^•−^. (**B**) The DMs are significantly associated with the antioxidant activities of FRAP.

As illustrated in Figure 11A, multiple organic acid DMs exhibit a significant relationship with DPPH•, FRAP, •OH, and O_2_^•−^. The correlation heatmap reveals that most DMs belonging to the carboxylic acids and derivatives class are positively correlated with DPPH•, •OH, and O_2_^•−^, such as glutamic acid, dimethylglycine, and Ll-2,6-diaminoheptanedioate. Furthermore, peptidomimetics DMs like L-carnosine also demonstrate a significant positive correlation with these three antioxidant indicators. The DMs that show a significant positive correlation with FRAP are L-arginine, proline, val-gln, n-acetyltryptophan, g-guanidinobutyrate, alanine, pro-leu, and his-ser (Figure 11B). The expression abundance of these DMs was consistent with the trend of DPPH•, FRAP, •OH, and O_2_^•−^ in different samples (Figure 12A,B). These DMs belong to the amino acids, peptides, and analogues subclass within the carboxylic acids and derivatives class. This suggests that amino acids play a major role in determining the antioxidant activity of Chinese cordyceps and its substitutes.

**Figure 13:**
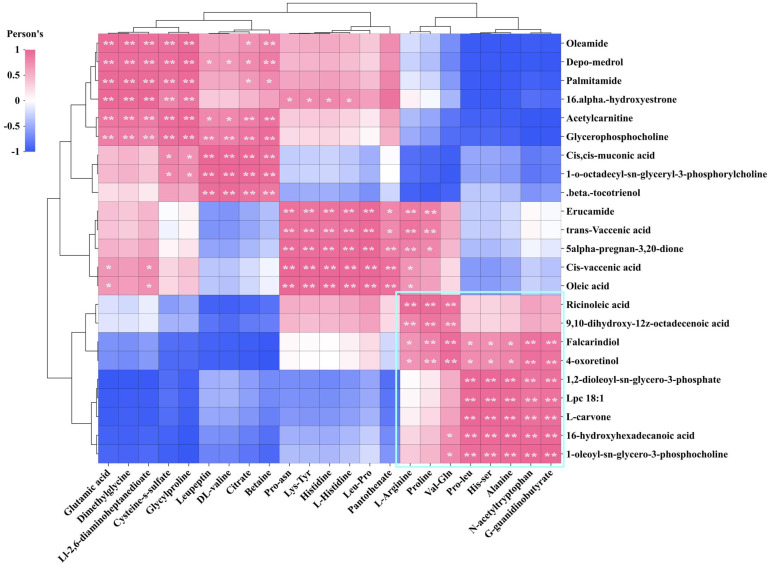
Correlation analysis of key lipid metabolites and key organic acids and derivatives. The vertical coordinate is key lipid metabolites; horizontal coordinate is key organic acids and derivatives. **, *p* values < 0.01. *, *p* values < 0.05.

We conducted a screening of DMs that were highly positively correlated with four antioxidant indicators, investigated potential correlations among these DMs, and generated a correlation heatmap. The analysis results indicate a significant positive correlation between lipid DMs and organic acid DMs that impact FRAP, as demonstrated by the DMS in the lower right corner. Additionally, their interdependent relationship has a notable enhancing or reducing effect on FRAP (Figure 13). However, these DMs had little effect on the antioxidant activities of DPPH•, •OH, and O_2_^•−^. L-arginine and proline showed a positive correlation with lipid DMs (e.g., erucamide, trans-vaccenic acid, cis-vaccenic acid, oleic acid, and 5alpha-pregnan-3,20-dione). This suggests that L-arginine and proline can impact the antioxidant activity of DPPH•, •OH, and O_2_^•−^. Conversely, changes in the levels of these lipid DMs will also influence the FRAP antioxidant capacity. 

### 3.5. KEGG Pathway Analysis

**Figure 14:**
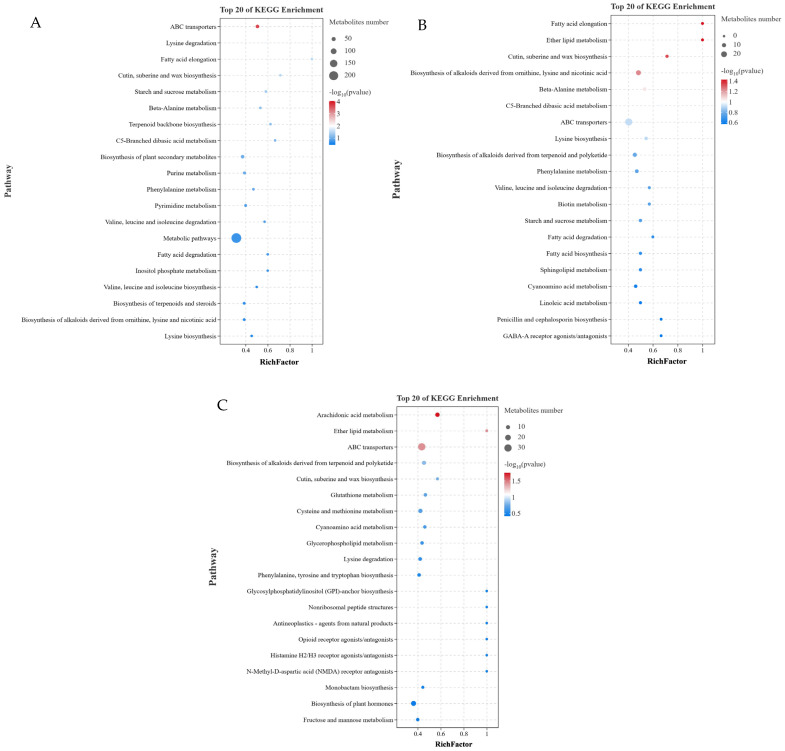
Enrichment analysis of DMs between GL, BL, and JSB using the KEGG database. The top 20 pathways with the lowest *p* value were mapped; the vertical coordinate is the pathway, the horizontal coordinate is the enrichment factor (the number of differential metabolites in this pathway divided by all the quantities in this pathway), the size represents the quantity, and the redder the color, the higher the *p* value. (**A**) GL vs. BL, (**B**) GL vs. JSB, (**C**) BL vs. JSB.

The KEGG pathway database was utilized to enhance the understanding of metabolic pathways based on the DM numbers in GL, BL, and JSB. The top 20 metabolic pathways with the highest enrichment rates were chosen for further analysis. The results showed that lysine biosynthesis, biosynthesis of alkaloids derived from ornithine, lysine and nicotinic acid, and biosynthesis of terpenoids and steroids were the top 3 pathways with significant differences in GL vs. BL (Figure 14A). The GABA-A receptor agonists/antagonists, penicillin and cephalosporin biosynthesis, and linoleic acid metabolism were the top 3 pathways with significant differences in GL vs. JSB (Figure 14B). The fructose and mannose metabolism, biosynthesis of plant hormones, and monobactam biosynthesis were the top 3 pathways with significant differences in BL vs. JSB (Figure 14C). These results indicated that GL, BL, and JSB may have different metabolite profiles, which may be linked to their antioxidant capacity. By screening, we found that ABC transporters and cutin, suberine and wax biosynthesis are metabolic pathways shared by the three samples. The metabolic pathway enrichment results showed that DMs were significantly enriched in ABC transporters but not significantly different among the three comparison groups (GL vs. BL, GL vs. JSB, and BL vs. JSB). The DMs of cutin, suberine and wax biosynthesis were significantly different in BL vs. JSB. In addition, the accumulation of fatty acid degradation and lysine biosynthesis pathways also affected the antioxidant activity of the three samples.

**Figure 15:**
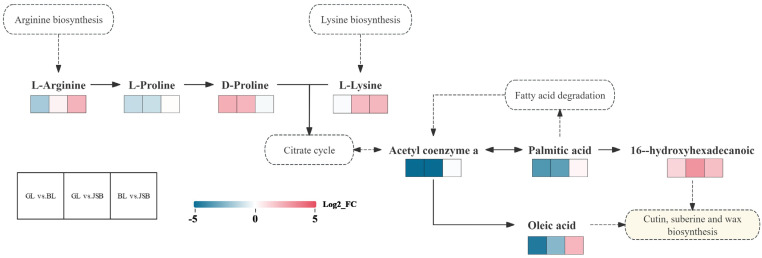
Overview of metabolic pathways mapped to possible regulation of key metabolites in pairwise comparisons of GL vs. BL, GL vs. JSB, and BL vs. JSB. The colored box of each metabolite indicates the corresponding log_2__FC value. Small red rectangles indicate significant upregulation of metabolites between groups; small blue rectangles indicate significant downregulation of metabolites between groups; small white rectangles represent insignificant differences between groups; solid arrows represent facilitation and dashed boxes indicate different metabolic pathways.

This study screens the most relevant metabolic pathways of overlapping differential metabolites based on pathway maps to facilitate an overview of changes in metabolic regulation (Figure 15). The results showed that the upregulation of L-arginine and L-lysine entering the citrate cycle may result in a significant increase in 16-hydroxyhexadecanoic content. Palmitic acid entered fatty acid degradation to promote the synthesis of acetyl coenzyme a and oleic acid. It is also possible that the 16-hydroxyhexadecanoic and oleic acid content is increased by the combined action of four pathways: arginine biosynthesis, lysine biosynthesis, citrate cycle, and fatty acid degradation. Then 16-hydroxyhexadecanoic and oleic acid enter cutin, suberine and wax biosynthesis, making the three samples have strong antioxidant capacity.

## 4. Discussion

Metabolomics is a grouping developed after genomics, transcriptomics, and proteomics, which is mainly based on high-throughput methods to qualitatively and quantitatively analyze the changes of small-molecule (molecular weight less than 1000 kDa) metabolites in samples and discover the correspondence between metabolites and physiological changes, then to find the potential biomarkers and enrich them to certain metabolic pathways [55,56]. Liquid chromatography-tandem mass spectrometry (LC-MS/MS) is an untargeted metabolomics analysis assay for the discovery of new biomarkers without the need for derivatization and is characterized by high throughput, broad coverage, low cost, and precise identification. However, untargeted metabolomics analysis may generate more false-positive data due to the lack of standards. In addition, the broad coverage of untargeted metabolomics technologies can detect data on unimportant substances that require complex data preprocessing and normalization to obtain more accurate qualitative metabolomics data.

Currently, LC-MS/MS analysis has been widely used to compare the metabolite profiles of cordyceps fungi from different geographical locations, species, and culture environments [57,58], and it can also be applied to analyze a certain class of metabolites (e.g., lipids, nucleosides, and proteins) in wild Chinese cordyceps and/or its substitutes [59,60,61]. However, the metabolic profiles of Chinese cordyceps, *P. hepiali*, and *O. sinensis* are unknown. Therefore, this study utilized multiple databases and employed LC-MS/MS technology in conjunction with multivariate statistical methods for qualitative analysis of the metabolites of the 3 samples, which shortened the detection time of the experiment while ensuring high sensitivity and obtaining more accurate qualitative metabolomics data.

The study revealed that the metabolites of GL, BL, and JSB can be categorized into 17 supercategories, including organic acids and derivatives, lipids and lipid-like molecules, organoheterocyclic compounds, and benzenoids. These findings align with previous research on the main types of metabolites [62]. Through HCA, it was determined that numerous DMs belonging to lipids and organic acids are significantly present in GL. For instance, the levels of L-glutamate and L-glutamine are found to be higher in GL compared to BL and JSB, aligning with the findings of Guo et al. [49]. L-theanine, a non-protein amino acid primarily sourced from tea [63], shares a structural similarity with glutamic acid. It plays a crucial role in enhancing immunity, safeguarding nerves, alleviating anxiety, and so on [64,65,66]. Furthermore, besides being identified in shiitake mushrooms [67], our study revealed the presence of L-theanine in BL as well. Interestingly, its abundance was found to be higher than GL and JSB, aligning with previous research findings [49]. D-mannitol, also known as Cordycepic acid, is a key active ingredient found in Chinese cordyceps and is commonly utilized for its antioxidant, anti-aging, hyperglycemia, renal failure, and arrhythmia properties [68,69]. We assessed the relative amounts of these metabolites based on their peak areas and found that D-mannitol was highly enriched in GL, followed by BL and JSB (refer to Table S1 for more information). Previous studies have indicated that BL has a higher Cordycepic acid content compared to JSB [70], while GL has a higher Cordycepic acid content than BL [71], aligning with our study findings.

Antioxidant activity results indicated that GL demonstrated strong DPPH free radical scavenging, hydroxyl free radical scavenging, and superoxide anion radical scavenging abilities, aligning with findings from previous studies [44,45]. Correlation analysis indicated a significant positive correlation between fatty acyls DMs and DPPH•, •OH, and O_2_^•−^. Interestingly, there was a decrease in the levels of these DMs in GL_vs_BL and GL_vs_JSB. Furthermore, the comparison between BL_vs_JSB suggested that the FRAP of JSB might be influenced by fatty acyls DMs, with a higher likelihood compared to GL and BL (more detailed information can be found in Table S1). The changes in the abundance of these DMs aligned with the variations in antioxidant activities across the samples, indicating that the antioxidant capacity of the samples could be impacted by the abundance and diversity of these DMs. Previous research has demonstrated a notable correlation between lipid DMS and antioxidant activity. Alterations in the levels of these metabolites could potentially have a more pronounced effect on antioxidant capacity, aligning with the findings of this study [72]. Wang et al. discovered that the high levels of glutamic acid and arginine present in Chinese cordyceps water extract contribute to its significant hydroxyl free radical scavenging capacity, DPPH free radical scavenging capacity, and reducing capability [73]. Glutamic acid, dimethylglycine, and Ll-2,6-diaminoheptanedioate were found to have significant impacts on the antioxidant activities of DPPH•, •OH, and O_2_^•^. Additionally, FRAP levels were influenced by amino acid DMs like val-gln, n-acetyltryptophan, and g-guanidinobutyrate. L-carnosine and pantothenate were found to have a positive correlation with Ll-2,6-diaminoheptanedioate, glutamic acid, and dimethylglycine while showing a negative correlation with val-gln, n-acetyltryptophan, and g-guanidinobutyrate. This suggests that they could potentially enhance the antioxidant activity of DPPH•, OH, and O_2_^•−^, while possibly decreasing FRAP levels. Research has demonstrated that plasmalogen, a derivative within the glycerophospholipids subclass, plays a crucial role in protecting cells from oxidative stress and maintaining them at normal levels; this function ultimately leads to a reduction in the occurrence of Alzheimer’s disease [74]; the addition of an appropriate amount of phospholipid to the diet of Juvenile Snakehead (Channa argus) can enhance the total antioxidant capacity of the liver and decrease lipid peroxidation damage [75]. The glycerophospholipids DMs that positively correlate with FRAP include Lpc 18:1, 1-oleoyl-sn-glycero-3-phosphocholine, and 1,2-dioleoyl-sn-glycero-3-phosphate. This suggests that the FRAP of the three samples may be influenced by these DMs. However, further in vivo experiments are required to confirm this.

Based on correlation analysis, it was observed that mainly amino acid DMs had a higher effect on the antioxidant activity of DPPH•, FRAP, OH, and O_2_^•−^, followed by fatty acyls DMs. Studies have shown that nucleosides and amino acids, isolated from Chinese cordyceps, protected against cyclophosphamide-induced myelosuppression in mice [76]. L-glutamic acid has benefits for human metabolism and without any associated risk of bodily accumulation [77,78,79]. Taurine, as a class of amino acid derivatives, not only has antioxidant, hypolipidemic, and sedative properties but also is important for infant growth and development [80]. Therefore, we could perform targeted metabolomics studies on amino acid compounds. Differential metabolites affecting the antioxidant activity of the three samples were clarified by qualitative and quantitative analysis of the detected amino acid compounds.

Distinct pathways for Chinese cordyceps and their surrogates were identified through KEGG pathway analysis. Our study demonstrates that acetyl coenzyme a, hexadecanoic acid, and palmitic acid are the primary lipid metabolites in the fatty acid degradation metabolic pathway, with higher relative abundances in the GL. Previous studies have demonstrated that wild Chinese cordyceps exhibit higher relative abundances of fatty acids [81], sphingolipids [82], and lipid metabolites [83], a finding that aligns with the results of our study. Flavonoids have been reported to possess a variety of pharmacological activities such as antioxidant, anti-inflammatory, antibacterial, hypoglycemic, and preventing and controlling respiratory diseases [84,85]. We identified 70 flavonoid metabolites in the biosynthesis of secondary metabolites, such as flavone, morin, and catechin, all of which were significantly upregulated in BL and JSB. In addition, we found that components with antioxidant [86] and anti-aging [87] effects (e.g., vanillic acid, ellagic acid) were significantly upregulated in BL. These findings suggest variations in the metabolites of Chinese cordyceps and their substitutes, offering insights into their potential pharmacological functions.

## 5. Conclusions

This study conducted an untargeted metabolomic analysis of Chinese cordyceps and its surrogates, revealing that the metabolites present in Chinese cordyceps, *P. hepiali*, and *O. sinensis* are primarily concentrated in organic acids and derivatives, lipids and lipid-like molecules, and organoheterocyclic compounds. Antioxidant activity analysis indicated that GL exhibited the highest DPPH free radical scavenging rate, hydroxyl free radical scavenging rate, and superoxide anion radical scavenging rate, while JSB demonstrated the highest FRAP total antioxidant capacity. Correlation analysis revealed significant positive correlations between fatty acyls and amino acid DMs with DPPH•, FRAP, •OH, and O_2_^•−^, while glycerophospholipids DMs also showed significant positive correlation with FRAP. Furthermore, KEGG pathway analysis highlighted the importance of the synthesis and metabolism of DMs in pathways such as cutin, suberine and wax biosynthesis in influencing the antioxidant activity of the three samples. The findings presented in this study shed light on the antioxidant capacity and metabolic characteristics of Chinese cordyceps and its substitutes, offering a deeper understanding of the metabolic markers influencing their antioxidant properties. These insights serve as a valuable reference for future research on their antioxidant activity and associated metabolites. However, relying solely on in vitro experiments and metabolomics to analyze the antioxidant activity of Chinese cordyceps and its substitutes has limitations. In future studies, we will conduct targeted metabolomics analysis of amino acid compounds based on the results of the present study, to investigate the effect of amino acid compounds on antioxidant activity through accurate qualitative and quantitative analysis. We might perform cell culture or animal experiments to test the in vivo antioxidant activity of Chinese cordyceps, *P. hepiali*, and *O. sinensis*, and then analyze them by combined metabolomic and transcriptomic analyses to understand their health effects and potential value. This may better provide theoretical support for the development of functional health foods from Chinese cordyceps and its substitutes.

## Figures and Tables

**Table 1:** Information of Chinese cordyceps, *O. sinensis* and *P. hepiali*.

Number	Species	Origin	Weight
GL	Chinese Cordyceps	Guoluo Tibetan Autonomous Prefecture, Qinghai Province	2 g
BL	*Ophiocordyceps sinensis*	Hangzhou Zhongmei Huadong Pharmaceutical Co., Ltd. (Hangzhou, China)	2 g
JSB	*Paecilomyces hepiali*	Jiangxi Jinshui Bao Pharmaceutical Co., Ltd. (Nanchang, China)	2 g

**Table 2:** The procedure of gradient separation.

Time (min)	Mobile Phase B
0–0.5	95%
0.5–7	65%
7–8	40%
8–9	40%
9–9.1	95%
9.1–12	95%

## Data Availability

The supporting data for the findings of this study are available from the corresponding authors upon reasonable request.

## Supplementary Materials

The following supporting information can be downloaded at: https://www.mdpi.com/article/10.3390/biology13090683/s1, Table S1: Up- or down-regulation relationships of 508 key metabolites in the three comparison groups with GL vs. BL, GL vs. JSB, and BL vs. JSB.

## Abbreviations

VC	Ascorbic acid
GL	Chinese cordyceps
CUR	Curtain gas
CAMERA	Collection of Algorithms of MEtabolite pRofile Annotation
DMs	Differential metabolites
DNA	Deoxyribonucleic acid
DP	Declustering potential
EDTA	Ethylenediaminetetraacetic acid
FRAP	FRAP total antioxidant capacity
HCA	Hierarchical Cluster Analysis
•OH	Hydroxyl free radical scavenging capacity
HMDB	Human Metabolome Database
LC-MS/MS	High-performance liquid chromatography-tandem mass spectrometry
ITS	Internal Transcribed Spacer
ISVF	IonSapary Voltage Floating
IDA	Information-dependent acquisition
KNN	K-Nearest Neighbor
KEGG	Kyoto Encyclopedia of Genes and Genomes database
R^2^Y	Model (for Y-variable dataset) Interpretability
Q^2^	Model predictability
R^2^	Model Interpretability
ESI−	Negative Electrospray Ionization
OPLS-DA	Orthogonal partial least squares discriminant analysis
BL	*Ophiocordyceps sinensis* (*O. sinensis*)
JSB	*Paecilomyces hepiali* (*P. hepiali*)
PVP	Polyvinyl pyrrolidone
PCA	Principal Component Analysis
ESI+	Positive Electrospray Ionization
PC1	Principal Component 1
PC2	Principal Component 2
QC	Quality control
RAPD	Random Amplified Polymorphic DNA
ROS	Reactive oxygen species
O_2_^•−^	Superoxide anion radical scavenging capacity
TIC	Total ion chromatograms
UHPLC	Ultra-high performance liquid chromatography
VIP	Variable importance
DPPH•	2,2-diphenyl-1-picrylhydrazyl radical scavenging ability
